# Incidence, Outcomes and Sex-Related Disparities in Pneumonia: A Matched-Pair Analysis with Data from Spanish Hospitals (2016–2019)

**DOI:** 10.3390/jcm10194339

**Published:** 2021-09-23

**Authors:** Jose M. de Miguel-Yanes, Ana Lopez-de-Andres, Rodrigo Jiménez-Garcia, Valentin Hernandez-Barrera, Javier de Miguel-Diez, David Carabantes-Alarcon, Napoleon Perez-Farinos, Julia Wärnberg

**Affiliations:** 1 Internal Medicine Department, Hospital General Universitario Gregorio Marañón, Instituto de Investigación Sanitaria Gregorio Marañón (IiSGM), Universidad Complutense de Madrid, 28040 Madrid, Spain; josemaria.demiguel@salud.madrid.org; 2 Department of Public Health & Maternal and Child Health, Faculty of Medicine, Universidad Complutense de Madrid, 28040 Madrid, Spain; rodrijim@ucm.es (R.J.-G.); dcaraban@ucm.es (D.C.-A.); 3 Preventive Medicine and Public Health Teaching and Research Unit, Health Sciences Faculty, Universidad Rey Juan Carlos, Alcorcón, 28922 Madrid, Spain; valentin.hernandez@urjc.es; 4 Respiratory Department, Hospital General Universitario Gregorio Marañón, Instituto de Investigación Sanitaria Gregorio Marañón (IiSGM), Universidad Complutense de Madrid, 28040 Madrid, Spain; javier.miguel@salud.madrid.org; 5 Instituto de Investigación Biomédica de Málaga (IBIMA), School of Medicine, Universidad de Málaga, 29071 Málaga, Spain; napoleon.perez@uma.es; 6 Instituto de Investigación Biomédica de Málaga (IBIMA), School of Health Sciences, Universidad de Málaga, 29071 Málaga, Spain; jwarnberg@uma.es

**Keywords:** community-acquired pneumonia, hospital-acquired pneumonia, ventilator-associated pneumonia, gender, sex differences, in-hospital mortality

## Abstract

(1) Background: the purpose of this study is to analyze the incidence and in-hospital mortality (IHM) of community-acquired pneumonia (CAP) needing hospital admission and hospital-acquired pneumonia (HAP) in Spain (2016–2019). (2) Methods: using the Spanish Register of Specialized Care-Basic Minimum Database, we estimated the incidence of CAP and HAP. We matched each woman with a man with an identical age, according to comorbidities. (3) Results: we analyzed 518,838 cases of CAP and 38,705 cases of HAP, and 5192 ventilator-associated HAPs (13.4%). The incidence of CAP increased over time in both men (from 384.5 to 449.8 cases/105 population) and women (from 244.9 to 301.2 cases/105 population). Men showed a 47% higher adjusted incidence of CAP than women. The incidence of HAP increased over time in both men (from 302.3 to 342.2 cases/105 population) and women (from 139.2 to 167.6 cases/105 population). Men showed a 98% higher adjusted incidence of HAP than women. IHM was higher in men admitted for CAP than in women (12.9% vs. 12.2%; *p* < 0.001), but not in men who developed HAP (28.9% vs. 28.0%; *p* = 0.107). Men admitted for CAP (OR: 1.13; 95% CI: 1.10–1.15) and men who developed HAP (OR: 1.05; 95% CI: 1.01–1.10) had higher IHM than women. (4) Conclusions: men had higher incidence rates of CAP and HAP than women. Men admitted for CAP and men who developed HAP had higher IHM than women.

## 1. Introduction

The burden imposed by community-acquired pneumonia (CAP) continues to be high in developed countries, especially among adults with underlying clinical risk conditions [1,2]. Incidence has seemingly been growing over the years, and the comorbidity of people affected by CAP needing hospital admission has been reported to be increasing across time [3].

Hospital-acquired pneumonia (HAP) is the second leading cause of nosocomial infection [4]. Important efforts have been put in place to reduce its morbimortality and associated health costs, and these have been mainly successful in the case of ventilator-associated pneumonia (VAP) [5]. However, VAP only represents a fraction of all HAPs. Indeed, voices have been raised to implement strategies to reduce the impact of non-ventilator-associated HAP (NV-HAP) [6]. Although updated guidelines to treat both conditions have been issued [7], some authors have argued that some of the recommendations given are not based on high-quality evidence [8].

Sex has been claimed to exert an influence on the incidence and outcomes of CAP, with a possible higher in-hospital mortality (IHM) among men [9]. In the case of HAP, apparently opposite findings have been published [10,11]. Biological, cultural, behavioral and socio-economic differences may be important determinants to explain the effect exerted by gender on the clinical management and outcomes of pneumonia [12]. However, sex differences in the provision of care resulting in delayed hospital admission, and consequently necessary care and use of hospital resources, has been reported among women [12]. Recent reviews on CAP have been found to be more severe in men than women, leading to higher mortality in males; this is more relevant in the older age groups [10,11,12]. Whilst a lot of attention is being paid to racial and ethnic differences in recent years, we perceive that research efforts should also focus on possible sex disparities in relevant, prevalent processes like pneumonia.

Our group has previously studied the influence of sex on both CAP and the two subtypes of HAP, namely VAP and NV-HAP [13]. However, the results of this work were somewhat limited by the short time period evaluated (2016–2017). Additionally, residual confounding could have biased the results to a certain extent, since no sex-matching analyses were performed.

Here, we aimed to compare the incidence, clinical characteristics, and in-hospital outcomes (length of hospital stay and in hospital mortality (IHM)) between women and men admitted for CAP or HAP to Spanish hospitals during the extended period of 2016–2019. In order to assess provider difference, we also compared the use of bronchial fibroscopy, computerized axial tomography of the thorax and dialysis. Possible differences in pathogen isolation according to sex were analyzed. 

We used propensity score matching (PSM) with the purpose of attenuating the baseline sex-related differences for the comparisons. We finally sought the variables associated with IHM for patients with either CAP or HAP according to sex.

## 2. Materials and Methods

### 2.1. Study Population

For our investigation, we used data from the Spanish Register of Specialized Care-Basic Minimum Database (RAE-CMBD) for the period between 1 January 2016 and 1 December 2019. Discharge diagnoses and procedures were coded based on the International Classification of Disease, Tenth Revision (ICD-10) (Table S1). Additional details on the RAE-CMBD can be found online [14].

We have used a similar methodology in previous research [13,15]. People older than 19 years admitted to the hospital for either CAP or who developed HAP during admission for another condition were included in the study. Only HAP patients with a “not present at admission” code who had a hospitalization lasting ≥48 h were included. HAP cases could be either VAP or NV-HAP. The study population was stratified according to sex.

### 2.2. Study Variables

We first calculated the trends in the incidence of CAP, HAP, NV-HAP and VAP among women and men. To calculate the incidences, we used the reports of the Spanish National Statistics Institute for the years 2016–2019 grouped by age and sex [16]. We assessed comorbidity with the Charlson Comorbidity Index (CCI), extracted with the methods for ICD-10-coded administrative databases [17,18]. Other study variables collected included age, comorbidities, use of oxygen prior to the index hospitalization, as well as diagnostic and therapeutic procedures, such as bronchial fibroscopy, computerized axial tomography of the thorax or dialysis (Table S1). The RAE-CMBD also allows us to identify patients who underwent any type of surgical procedure during their hospital admission. The main outcomes were IHM and length of hospital stay.

Regarding pathogen isolation, we could identify the following codes as confirmed by the laboratory: *Aspergillus*, *Candida*, *Escherichia coli*, *Haemophilus influenzae*, *Klebsiella pneumoniae*, *Legionella*, non-specified *Streptococcus*, *Pseudomonas aeruginosa*, other Gram-negative bacteria, *Staphylococcus aureus*, *Streptococcus pneumoniae*, *Influenza* virus, and “other virus” (Table S1).

### 2.3. Matching Method

We used a PSM method to create subpopulations more amenable to comparisons based on their baseline conditions [19]. The PSM was conducted using a multivariable logistic regression in which the matching variables were age, sex and comorbid conditions present at admission. 

### 2.4. Statistical Analysis

We evaluated the incidences using Poisson regression models adjusted for age and sex when required, expressed as incidence rate ratios (IRRs) with 95% confidence intervals (95% CIs). We reported means with standard deviations (SDs) or medians with interquartile ranges (IQRs) as descriptive statistics for continuously distributed variables, and absolute frequencies and proportions for categorical variables. We compared the continuously distributed variables with the t test or the Mann-Whitney test, and the categorical variables with the chi-square test. We used the McNemar’s test and a paired t test to compare the study subgroups after PSM [19].

We used multivariable logistic regression analyses to identify the variables independently associated with IHM. We constructed models separately for men and women. Finally, we analyzed the effect of sex in the entire database. The results we obtained are expressed as odds ratios (ORs) with their 95% CIs.

The software used for matching and statistical analysis was Stata version 14 (Stata, College Station, TX, USA). We set statistical significance at a two-sided *p*-value of < 0.05. 

### 2.5. Ethics

The RAE-CMBD is owned by the Spanish Ministry of Health and can be accessed upon request [20]. This registry is anonymized and under public access, which means that according to Spanish legislation, approval by an ethics committee can be waived.

## 3. Results

During the 4-year period of 2016–2019, 518,838 cases of CAP (58.9% men) (Table 1), and 38,705 cases of HAP (65.4% men) (Table 2) were coded. There were 5192 cases of VAP (representing 13.4% of HAPs), also with a preponderance of the male sex (71.1%) (Table 2).

### 3.1. Incidence of Hospital Admission for Pneumonia and Hospital-Acquired Pneumonia According to Sex

The incidence of hospital admission for CAP increased over time in both men (from 384.5 to 449.8 cases/10^5^ population) and women (from 244.9 to 301.2 cases/10^5^ population) (both *p* < 0.001). The increasing incidences were mainly driven by the age groups 60–74 and ≥85 years in both sexes (Table 1). Using Poisson’s regression analyses, men showed a 47% higher adjusted incidence than women (IRR = 1.47; 95% CI: 1.45–1.50).

The incidence of people who developed HAP also increased over time in both men (from 302.3 to 342.2 cases/10^5^ population) and women (from 139.2 to 167.6 cases/10^5^ population) (both *p* < 0.001). Increasing incidences were seen for both VAP (from 39.9 to 56.1 cases/10^5^ population in men, *p* < 0.001, and from 16.6 to 19.9 cases/10^5^ population in women, *p* = 0.002), and NV-HAP (from 262.4 to 286.1 cases/10^5^ population in men, and from 122.6 to 147.7 cases/10^5^ population in women (both *p* < 0.001)). Again, the increasing incidences were mainly driven by the age groups 60–74 and ≥85 years in both sexes (Table 2). Men showed higher adjusted incidences than women: for HAP, IRR = 1.98 (95% CI: 1.97–1.99); for VAP, IRR = 2.21 (95% CI: 2.15–2.27), and for NV-HAP, IRR = 1.83 (95% CI: 1.80–1.86).

Over time, only people admitted to the hospital for CAP were older. Whilst comorbidity increased over time, the length of hospital stay remained relatively stable (Table 1 and Table 2). We saw significant decreases in IHM in people admitted for CAP, but not in people who developed HAP.

According to Tables S2 and S3, *Klebsiella pneumoniae* and *Legionella pneumophila* were increasingly coded in women admitted for CAP, whereas the *Influenza* virus was increasingly coded in men. The category “other virus” became more frequently coded in both sexes. Tables S4 and S5 show the distribution of pathogens in women and men hospitalized with CAP or who developed HAP, before and after PSM.

### 3.2. Clinical Characteristics and Outcomes for Women and Men Admitted to the Hospital for CAP

Before PSM, women admitted for CAP were older than men, but had lower overall comorbidity, except for heart failure, dementia, and rheumatoid disease codes (more frequent among women) (Table 3). The differences in baseline characteristics between sexes were partially attenuated by PSM. Men underwent bronchial fibroscopy, chest computerized tomography, dialysis, invasive or non-invasive mechanical ventilation and any surgery more often than women. IHM was higher in men than in women admitted for CAP (12.5% vs. 12.2% before PSM (*p* = 0.001); and 12.9% vs. 12.2% (a 5.7% higher relative risk among men) after PSM (*p* < 0.001)).

### 3.3. Clinical Characteristics and Outcomes for Women and Men Who Developed HAP

Before PSM, women who developed HAP were older than men, but had lower overall comorbidity, except for heart failure, dementia, and rheumatoid disease codes (more frequent among women). The differences between sexes were not significant for cerebrovascular disease, type 2 diabetes mellitus and renal disease (Table 4). PSM equalized ages between men and women, but only partially attenuated other differences in the baseline characteristics. Men underwent bronchial fibroscopy, chest computerized tomography, dialysis, and any surgery more often than women. The difference in IHM seen before PSM (29.2% among men vs. 28.0% among women; *p* = 0.01) became non-significant after PSM (28.9% among men vs. 28.0% among women, a 3.2% higher relative risk among men (*p* = 0.107)).

### 3.4. Multivariable Analysis of Variables Associated with IHM for Women and Men Admitted to the Hospital for CAP

As can been seen in Table 5, the risk of dying during hospital admission for CAP increased with age and each comorbidity included in the model that showed statistical significance, except for prevalent chronic obstructive pulmonary disease (COPD) (OR: 0.76 (95% CI: 0.73–0.80)), and diabetes (OR: 0.91 (95% CI: 0.89–0.93)). Both non-invasive and invasive mechanical ventilation were associated with a higher IHM, irrespectively of sex. Over time, IHM decreased significantly in both men and women admitted for CAP. After accounting for potential confounders, men had a higher IHM than women in people admitted for CAP (OR: 1.13 (95% CI: 1.10–1.15)).

### 3.5. Multivariable Analysis of Variables Associated with IHM for Women and Men Who Developed HAP

In Table 6, we can see the comorbidities associated with a higher IHM in men and women who developed HAP during a hospital admission. IHM increased with age, but having undergone a surgical procedure was associated with a lower IHM in people who developed HAP during the same hospital admission (OR: 0.74 (95% CI: 0.70–0.78)). Over time, IHM was significantly lower only in women. VAP was associated with a higher IHM in both sexes (in the overall population, OR: 1.95 (95% CI: 1.82–2.10)). After accounting for potential confounders, men had a higher IHM than women who developed HAP during a hospital admission (OR: 1.05 (95% CI: 1.01–1.10)).

## 4. Discussion

Here, we found increasing incidences of CAP with hospital admission and HAP—both VAP and NV-HAP—from 2016 to 2019 in Spanish hospitals. Men showed higher adjusted incidences than women for all kinds of pneumonia. We saw significant decreases in IHM over time in people admitted for CAP, and in women who developed HAP, but not in men who developed HAP. Finally, men had higher IHM than women for both CAP and HAP in the multivariate analyses.

We could see increasing incidences of CAP needing hospital admission over time. Although this finding could simply reflect less restrictive admission criteria for CAP in an older, more comorbid population instead of actual yearly increases in the incidence of CAP, additional evidence supports true increases in the incidence of CAP [21]. However, the COVID-19 pandemic onset will probably represent an inflection point for the incidence of CAP cases unrelated to SARS-CoV-2. Indeed, during the first waves of the COVID-19 pandemic, lower rates of admission for CAP have been reported, mainly in milder cases [22]. 

Older research had previously shown relatively similar incidence rates for CAP, with higher rates in men with clinical risk factors [23] and with advancing age [24]. Perhaps a lower compliance with influenza vaccination in recent years in our country can provide an additional explanation for our findings [25]. As far as incidence is concerned, we believe that through the PSM method followed in this study, we contribute valuable data thanks to the attenuation of the residual confounding that has precluded drawing more definite conclusions in previous studies. 

We also confirmed increasing incidences of VAP and NV-HAP. There are large discrepancies in the incidence rates reported for VAP in the literature, which partially obey the different criteria used for its diagnosis, the differences in the microbiological sampling methods [26], and eventually the disparities in the populations studied (e.g., surgical vs. non-surgical patients) [27]. We found that men had higher incidences than women, accordingly to previous research [28]. Yet, the most important risk factor for VAP is likely the underlying medical conditions of the patients, both the severity of the current illness and the prevalent coexisting medical conditions [29]. 

As regards NV-HAP, the identification of patients with this condition presents a challenge for clinicians due to the dispersion of cases throughout all clinical areas of the hospital [30]. In fact, NV-HAP can develop in patients admitted with no clinical risk factors to develop pneumonia other than hospital admission [31]. These circumstances make it difficult to reduce the incidence of NV-HAP in everyday clinical practice, especially in bedridden patients with a high risk for bronchial aspiration [32]. Additional risk factors are age, disturbance of consciousness, CCI score, antibiotic use and glucocorticoid use [33].

The US National Vital Statistics System has shown decreasing mortality rates for pneumonia during the period 2016–2019 [34]. In our multivariate analyses, IHM decreased over time for all types of pneumonia, except for men who developed HAP, who nonetheless showed a non-significant trend towards a lower mortality rate. Previous research had described higher mortality rates among men who developed NV-HAP than among women [35]. In our study we observed that men had higher IHM than women overall. On the one hand, men received more diagnostic and therapeutic procedures when admitted for CAP or whenever they developed HAP, but on the other hand had a higher number of comorbidities. Perhaps a worse clinical situation during admission prompted the indication of a higher number of procedures in a population with an a priori higher probability of death during the hospital stay. Alternatively, gender differences in clinical presentation, subjective impressions of illness severity or objective severity scores could have translated into distinct behaviors in the clinical management of sick men and women on behalf of the treating physicians [36]. Indeed, our group has previously reported a sex gap in the indication of non-invasive vs. invasive mechanical ventilation in people admitted to Spanish hospitals [37]. In this study, IHM increased linearly among women admitted for CAP, but men older than 84 showed lower mortality rates than men aged 75 to 84 years. This phenomenon was even more remarkable in the case of HAP, with a relatively low IHM among very old men (14.36%), perhaps reflecting some sort of advantage for having overcome competing risks, or a distinctive biological response in male patients who have lived beyond 84 years old. 

Both non-invasive and invasive mechanical ventilation were associated with a higher IHM, irrespectively of sex. These should not be interpreted as causal facts, but rather as significant associations found in an observational study where confounding by indication may be underlying. Prevalent COPD and diabetes were associated with a lower mortality risk in the population admitted for CAP in the multivariate analyses after extensively accounting for potential confounding variables. We had previously detected lower IHM in COPD patients admitted for CAP [38], and in people with diabetes admitted for CAP [39,40], using a similar database for the period 2004–2013. We might speculate that patients diagnosed with any of these two conditions were admitted to the hospital earlier that people unaffected. In a meta-analysis published by Jiang et al., COPD was associated with a lower IHM in patients admitted for CAP. In a similar fashion, some authors have claimed that hyperglycemia may pose a higher IHM, rather than prevalent diabetes itself [41].

We noticed that having undergone a surgical procedure was associated with a lower IHM in people who developed HAP during the same hospital admission. We had previously reported mortality rates around 30% in people who developed postoperative pneumonia in Spain from 2001–2015 [42]. Arguably, both people admitted to the hospital for a surgical procedure, and people admitted to the hospital for other reasons who underwent a surgical procedure, had a better performance and health status than people who did not undergo any surgical procedure. Although some risk factors are not modifiable, we could act on surgical-specific modifiable factors, such as oral bacterial load or smoking habits, in an attempt to reduce the incidence of HAP in surgical patients if feasible before elective surgery [43].

Finally, we detected that people who developed VAP had a higher IHM than people who developed NV-HAP among both sexes (in the overall population, a 95% higher IHM), in accordance with previous reports [44], probably due to the severity and type of underlying disease leading to the indication of mechanical ventilation, whilst not being driven by the procedure itself. The European Respiratory Society has launched a project to develop new international guidelines for HAP and VAP due to their unacceptably high mortality rates [7]. The reality is that VAP still has a high attributable mortality, that is, the percentage of deaths that would not have occurred in the absence of the infection [45].

As the main strengths of our study, we can put forward its large sample size, with data from over 557,000 episodes of pneumonia, the widespread coverage of the Spanish population by the RAE-CMBD (>95% of all hospital admissions) and the standardized methodology, which we have previously used [15]. Yet, we should point out several limitations. Our data source is an administrative database supported by the information that physicians keep in the discharge report, which also depends on manual coding on behalf of the administrative staff. Despite a pair-matching process that most likely contributed to attenuate sex-related differences in baseline characteristics and clinical variables, a complete elimination of residual confounding is difficult to achieve in observational case–control studies. Although baseline conditions were vastly accounted for, we could not adjust for clinical severity at presentation with well-established, validated scores. Nevertheless, potential indirect markers of acute severity, such as need of mechanical ventilation or dialysis, were included in the models. We have extensively discussed elsewhere about the possible inaccuracy of CAP and HAP coding in administrative databases [13].

We settled stringent criteria for the definition of HAP, seeking specificity. Thus, people discharged home after an admission for other reasons who were readmitted due to pneumonia shortly after being sent home were not included in the study. Moreover, in our study, anonymity precludes the extraction of some specific pieces of information (i.e., people who moved from one hospital to another would appear twice), and the same patient could contribute with more than one episode of CAP or HAP. However, the information bias that results in a multiple admission of a patient would, in our opinion, be non-differential as it would affect men and women with the same intensity. Therefore, the main conclusions of our investigation would not be affected. Detailed information on some clinical variables, such as antibiotic or corticoid therapy, and others, were not available. Furthermore, according to the RAE-CMBD methodology, in the secondary diagnosis position only those conditions, risk factors or diseases that have directly affected the length of hospital stay, the diagnosis or therapeutic procedures conducted during the hospitalization, or the mortality should be included. Therefore, smoking status, which is an important confounding factor with gender imbalance, is not collected in our database and could not be analyzed.

Finally, we had no access to socio-economic data and such information could improve the value of our results. 

## 5. Conclusions

In summary, both CAP needing hospital admission and HAP are problems of the first magnitude in our health system. We found higher incidences and IHM among men than among women for every type of pneumonia studied. Future research efforts should focus on identifying possible reasons that explain sex differences in order to reduce them.

## Figures and Tables

**Table 1:** Incidence, clinical characteristics and in-hospital outcomes of patients hospitalized with community-acquired pneumonia (CAP) in Spain from 2016 to 2019 according to sex.

		2016	2017	2018	2019	*p*-Value for Trends
*N* (incidence per 100,000 inhabitants)	Men	68,300 (384.48)	74,352 (418.55)	82,851 (465.39)	80,077 (449.81)	<0.001
	Women	46,048 (244.86)	51,634 (274.57)	58,860 (312.53)	56,716 (301.15)	<0.001
Age, mean (SD)	Men	72.87 (15.18)	73.96 (14.69)	73.61 (14.86)	73.5 (15.04)	<0.001
	Women	75.01 (16.6)	76.5 (15.68)	76.12 (15.91)	75.94 (16.18)	<0.001
20–59 years, *n* (%)	Men	12,420 (18.18)	11,984 (16.12)	13,975 (16.87)	13,800 (17.23)	<0.001
	Women	8382 (18.2)	7817 (15.14)	9296 (15.79)	9223 (16.26)	<0.001
60–74 years, *n* (%)	Men	18,197 (26.64)	19,819 (26.66)	22,480 (27.13)	21,799 (27.22)	<0.001
	Women	8773 (19.05)	9925 (19.22)	11,739 (19.94)	11,585 (20.43)	<0.001
75–84 years, *n* (%)	Men	21,552 (31.55)	23,321 (31.37)	24,967 (30.13)	23,275 (29.07)	<0.001
	Women	12,733 (27.65)	14,084 (27.28)	15,442 (26.24)	14,042 (24.76)	<0.001
≥85 years, *n* (%)	Men	16,131 (23.62)	19,228 (25.86)	21,429 (25.86)	21,203 (26.48)	<0.001
	Women	16,160 (35.09)	19,808 (38.36)	22,383 (38.03)	21,866 (38.55)	<0.001
CCI, mean (SD)	Men	2.24 (2.08)	2.35 (2.08)	2.34 (2.11)	2.44 (2.19)	<0.001
	Women	1.71 (1.73)	1.81 (1.77)	1.79 (1.77)	1.87 (1.84)	<0.001
LOHS, median (IQR)	Men	7 (8)	7 (7)	7 (7)	7 (7)	<0.001
	Women	7 (7)	7 (7)	7 (7)	7 (7)	<0.001
IHM, *n* (%)	Men	8686 (12.72)	9412 (12.66)	10,363 (12.51)	9606 (12)	<0.001
	Women	5747 (12.48)	6511 (12.61)	7134 (12.12)	6534 (11.52)	<0.001

CCI: Charlson comorbidity index; LOHS: Length of hospital stay; IHM: In-hospital mortality.

**Table 2:** Incidence, clinical characteristics and in-hospital outcomes of patients who developed hospital-acquired pneumonia (HAP) in Spain from 2016 to 2019 according to sex.

		2016	2017	2018	2019	*p*-Value for Trends
*N* (incidence of HAP per 100,000 subjects hospitalized)	Men	5626 (302.27)	6162 (316.83)	6779 (349.68)	6740 (342.16)	<0.001
	Women	2936 (139.18)	3252 (148.38)	3579 (166.96)	3631 (167.6)	<0.001
*N* (incidence of NV-HAP per 100,000 subjects hospitalized)	Men	4884 (262.4)	5314 (273.23)	5781 (298.2)	5635 (286.06)	<0.001
	Women	2585 (122.54)	2913 (132.91)	3202 (149.38)	3199 (147.66)	<0.001
*N* (incidence of VAP per 100,000 subjects hospitalized)	Men	742 (39.87)	848 (43.6)	998 (51.48)	1105 (56.1)	<0.001
	Women	351 (16.64)	339 (15.47)	377 (17.59)	432 (19.94)	0.002
Age, mean (SD)	Men	69.03 (14.7)	69.43 (15.14)	69.47 (14.72)	68.81 (15.07)	0.029
	Women	72.02 (16.03)	72.33 (15.93)	73.09 (15.98)	72.58 (15.73)	0.047
20–59 years, *n* (%)	Men	1367 (24.3)	1491 (24.2)	1603 (23.65)	1648 (24.45)	<0.001
	Women	633 (21.56)	680 (20.91)	702 (19.61)	755 (20.79)	0.001
60–74 years, *n* (%)	Men	1924 (34.2)	2013 (32.67)	2312 (34.11)	2376 (35.25)	<0.001
	Women	750 (25.54)	834 (25.65)	895 (25.01)	969 (26.69)	<0.001
75–84 years, *n* (%)	Men	1595 (28.35)	1724 (27.98)	1855 (27.36)	1766 (26.2)	0.001
	Women	849 (28.92)	898 (27.61)	990 (27.66)	939 (25.86)	0.008
≥85 years, *n* (%)	Men	740 (13.15)	934 (15.16)	1009 (14.88)	950 (14.09)	<0.001
	Women	704 (23.98)	840 (25.83)	992 (27.72)	968 (26.66)	<0.001
CCI, mean (SD)	Men	2.73 (2.28)	2.75 (2.23)	2.76 (2.26)	2.83 (2.28)	0.107
	Women	2.3 (1.98)	2.28 (1.99)	2.39 (2.04)	2.47 (2.12)	<0.001
LOHS, median (IQR)	Men	24 (29)	24 (28)	24 (27)	25 (29)	0.492
	Women	23 (26)	22 (26)	23 (26)	23 (27)	0.425
IHM, *n* (%)	Men	1631 (28.99)	1843 (29.91)	2004 (29.56)	1911 (28.35)	0.223
	Women	871 (29.67)	895 (27.52)	996 (27.83)	984 (27.1)	0.116

HAP: hospital-acquired pneumonia; NV-HAP: non-ventilator-associated hospital-acquired pneumonia; VAP: ventilator-associated pneumonia; CCI: Charlson comorbidity index; LOHS: length of hospital stay; IHM: in-hospital mortality.

**Table 3:** Distribution of study covariates and hospital outcomes for women and men hospitalized with community-acquired pneumonia (CAP) in Spain (2016–2019), before and after propensity score matching (PSM).

	BEFORE PSM			AFTER PSM		
	Men	Women	*p*-Value	Men	Women	*p*-Value
*N* (incidence per 100,000 inhabitants)	305,580 (429.59)	213,258 (283.3)	<0.001	213,258 (NA)	213,258 (NA)	
Age, mean (SD)	73.5 (14.95)	75.92 (16.09)	<0.001	73.55 (16.21)	75.92 (16.09)	<0.001
20–59 years old, *n* (%)	52,179 (17.08)	34,718 (16.28)	<0.001	44,145 (20.7)	34,718 (16.28)	<0.001
60–74 years old, *n* (%)	82,295 (26.93)	42,022 (19.7)	<0.001	52,952 (24.83)	42,022 (19.7)	<0.001
75–84 years old, *n* (%)	93,115 (30.47)	56,301 (26.4)	<0.001	59,259 (27.79)	56,301 (26.4)	<0.001
≥85 years old, *n* (%)	77,991 (25.52)	80,217 (37.62)	<0.001	56,902 (26.68)	80,217 (37.62)	<0.001
CCI, mean (SD)	2.35 (2.12)	1.8 (1.78)	<0.001	1.93 (1.95)	1.8 (1.78)	<0.001
Myocardial infarction, *n* (%)	19,232 (6.29)	6039 (2.83)	<0.001	8029 (3.76)	6039 (2.83)	<0.001
Congestive heart failure, *n* (%)	62,570 (20.48)	54,436 (25.53)	<0.001	43,264 (20.29)	54,436 (25.53)	<0.001
Peripheral vascular disease, *n* (%)	22,400 (7.33)	5158 (2.42)	<0.001	4692 (2.2)	5158 (2.42)	<0.001
Cerebrovascular disease, *n* (%)	20,763 (6.79)	12,056 (5.65)	<0.001	13,073 (6.13)	12,056 (5.65)	<0.001
Dementia, *n* (%)	22,475 (7.35)	24,188 (11.34)	<0.001	18,302 (8.58)	24,188 (11.34)	<0.001
COPD, *n* (%)	79,200 (25.92)	16,322 (7.65)	<0.001	24,549 (11.51)	16,322 (7.65)	<0.001
T2DM, *n* (%)	86,761 (28.39)	53,436 (25.06)	<0.001	53,413 (25.05)	53,436 (25.06)	0.935
Rheumatoid disease, *n* (%)	5591 (1.83)	7966 (3.74)	<0.001	5066 (2.38)	7966 (3.74)	<0.001
Peptic ulcer, *n* (%)	2089 (0.68)	858 (0.4)	<0.001	999 (0.47)	858 (0.4)	0.001
Mild, moderate/severe liver disease, *n* (%)	22,020 (7.21)	9142 (4.29)	<0.001	12,599 (5.91)	9142 (4.29)	<0.001
Hemiplegia or paraplegia, *n* (%)	2744 (0.9)	1514 (0.71)	<0.001	2033 (0.95)	1514 (0.71)	<0.001
Renal disease, *n* (%)	58,964 (19.3)	36,933 (17.32)	<0.001	36,102 (16.93)	36,933 (17.32)	0.001
Cancer and metastatic cancer, *n* (%)	49,633 (16.24)	19,491 (9.14)	<0.001	30,892 (14.49)	19,491 (9.14)	<0.001
AIDS, *n* (%)	3685 (1.21)	1468 (0.69)	<0.001	2769 (1.3)	1468 (0.69)	<0.001
Undergone surgery, *n* (%)	9304 (3.04)	4937 (2.32)	<0.001	6590 (3.09)	4937 (2.32)	<0.001
Bronchial fibroscopy, *n* (%)	3975 (1.3)	1929 (0.9)	<0.001	2847 (1.34)	1929 (0.9)	<0.001
Computerized axial tomography of thorax, *n* (%)	20,140 (6.59)	10,887 (5.11)	<0.001	14,152 (6.64)	10,887 (5.11)	<0.001
Dialysis, *n* (%)	3951 (1.29)	1639 (0.77)	<0.001	2608 (1.22)	1639 (0.77)	<0.001
Oxygen prior to hospitalization, *n* (%)	21,244 (6.95)	11,932 (5.6)	<0.001	9278 (4.35)	11,932 (5.6)	<0.001
Non-invasive mechanical ventilation, *n* (%)	8957 (2.93)	5688 (2.67)	<0.001	5286 (2.48)	5688 (2.67)	<0.001
Invasive mechanical ventilation, *n* (%)	8675 (2.84)	4248 (1.99)	<0.001	6224 (2.92)	4248 (1.99)	<0.001
LOHS, median (IQR)	7 (7)	7 (7)	0.353	7 (7)	7 (7)	0.553
IHM, *n* (%)	38,067 (12.46)	25,926 (12.16)	0.001	27,531 (12.91)	25,926 (12.16)	<0.001

CCI: Charlson comorbidity index; COPD: chronic obstructive pulmonary disease; T2DM: type 2 diabetes mellitus; AIDS: Acquired immune deficiency syndrome; LOHS: length of hospital stay; IHM: in-hospital mortality. NA: not applicable.

**Table 4:** Incidence distribution of study covariates and hospital outcomes for women and men who developed hospital-acquired pneumonia (HAP) in Spain (2016–2019), before and after propensity score matching (PSM).

	BEFORE PSM			AFTER PSM		
	Men	Women	*p*-Value	Men	Women	*p*-Value
*N* (incidence of HAP per 100,000 subjects hospitalized)	24,357 (315.73)	13,398 (155.59)	<0.001	13,398 (NA)	13,398 (NA)	
*N* (incidence of NV-HAP per 100,000 subjects hospitalized)	21,614 (280.17)	11,899 (138.18)	<0.001	11,899 (NA)	11,899 (NA)	
*N* (incidence of VAP per 100,000 subjects hospitalized)	3693 (47.87)	1499 (17.41)	<0.001	1499 (NA)	1499 (NA)	
Age, mean (SD)	69.19 (14.91)	72.53 (15.92)	<0.001	72.46 (13.28)	72.53 (15.92)	0.655
20–59 years old, *n* (%)	6109 (24.14)	2770 (20.67)	<0.001	2297 (17.14)	2770 (20.67)	<0.001
60–74 years old, *n* (%)	8625 (34.08)	3448 (25.74)	<0.001	4430 (33.06)	3448 (25.74)	<0.001
75–84 years old, *n* (%)	6940 (27.42)	3676 (27.44)	0.977	4195 (31.31)	3676 (27.44)	<0.001
≥85 years old, *n* (%)	3633 (14.36)	3504 (26.15)	<0.001	2476 (18.48)	3504 (26.15)	<0.001
CCI, mean (SD)	2.77 (2.26)	2.36 (2.04)	<0.001	2.12 (2.01)	2.36 (2.04)	<0.001
Myocardial infarction, *n* (%)	2429 (9.6)	655 (4.89)	<0.001	278 (2.07)	655 (4.89)	<0.001
Congestive heart failure, *n* (%)	5632 (22.25)	3908 (29.17)	<0.001	3331 (24.86)	3908 (29.17)	<0.001
Peripheral vascular disease, *n* (%)	2656 (10.5)	630 (4.7)	<0.001	178 (1.33)	630 (4.7)	<0.001
Cerebrovascular disease, *n* (%)	3862 (15.26)	2076 (15.49)	0.543	2263 (16.89)	2076 (15.49)	0.002
Dementia, *n* (%)	1020 (4.03)	876 (6.54)	<0.001	793 (5.92)	876 (6.54)	0.036
COPD, *n* (%)	4194 (16.57)	780 (5.82)	<0.001	1032 (9.20)	780 (5.82)	<0.001
T2DM, *n* (%)	6158 (24.33)	3184 (23.76)	0.214	3050 (22.76)	3184 (23.76)	0.053
Rheumatoid disease, *n* (%)	338 (1.34)	458 (3.42)	<0.001	297 (2.22)	458 (3.42)	<0.001
Peptic ulcer, *n* (%)	548 (2.17)	241 (1.8)	0.015	227 (1.69)	241 (1.8)	0.514
Mild, moderate/severe liver disease, *n* (%)	2749 (10.86)	897 (6.7)	<0.001	499 (3.72)	897 (6.7)	<0.001
Hemiplegia or paraplegia, *n* (%)	1574 (6.22)	772 (5.76)	0.073	856 (6.39)	772 (5.76)	0.032
Renal disease, *n* (%)	4284 (16.93)	2316 (17.29)	0.373	2132 (15.91)	2316 (17.29)	0.003
Cancer and metastatic cancer *n* (%)	6557 (25.91)	2562 (19.12)	<0.001	2592 (19.35)	2562 (19.12)	0.642
AIDS, *n* (%)	232 (0.92)	57 (0.43)	<0.001	80 (0.6)	57 (0.43)	0.049
Undergone surgery, *n* (%)	12,833 (50.71)	6051 (45.16)	<0.001	6449 (48.13)	6051 (45.16)	<0.001
Bronchial fibroscopy, *n* (%)	812 (3.21)	320 (2.39)	<0.001	376 (2.81)	320 (2.39)	0.031
Computerized axial tomography of thorax, *n* (%)	2130 (8.42)	894 (6.67)	<0.001	1064 (7.94)	894 (6.67)	<0.001
Dialysis, *n* (%)	1863 (7.36)	679 (5.07)	<0.001	893 (6.67)	679 (5.07)	<0.001
Oxygen prior to hospitalization *n* (%)	698 (2.76)	359 (2.68)	0.652	255 (1.9)	359 (2.68)	<0.001
LOHS, median (IQR)	24 (29)	23 (26)	<0.001	24 (28)	23 (26)	<0.001
IHM, *n* (%)	7389 (29.2)	3746 (27.96)	0.010	3865 (28.85)	3746 (27.96)	0.107

HAP: hospital-acquired pneumonia; NV-HAP: non-ventilator-associated hospital-acquired pneumonia; VAP: ventilator-associated pneumonia; CCI: Charlson comorbidity index; COPD: chronic obstructive pulmonary disease; T2DM: type 2 diabetes mellitus; AIDS: Acquired immune deficiency syndrome; LOHS: length of hospital stay; IHM: in-hospital mortality. NA: not applicable.

**Table 5:** Multivariable analysis of factors associated with in-hospital mortality during admission for community-acquired pneumonia (CAP), according to sex.

	MEN	WOMEN	BOTH
	OR (95% CI)	OR (95% CI)	OR (95% CI)
20–59 years old	1	1	1
60–74 years old	1.77 (1.69–1.85)	1.56 (1.46–1.67)	1.77 (1.70–1.85)
75–84 years old	2.62 (2.50–2.74)	2.95 (2.77–3.14)	2.89 (2.78–3.01)
≥85 years old	4.48 (4.28–4.7)	5.51 (5.18–5.85)	5.19 (4.99–5.4)
Myocardial infarction	1.09 (1.04–1.14)	1.27 (1.19–1.37)	1.18 (1.13–1.24)
Congestive heart failure	1.34 (1.31–1.38)	1.29 (1.25–1.33)	1.31 (1.28–1.34)
Cerebrovascular disease	1.45 (1.40–1.51)	1.60 (1.52–1.68)	1.52 (1.47–1.58)
Dementia	2.09 (2.02–2.17)	1.94 (1.87–2.01)	2.03 (1.98–2.09)
COPD	0.73 (0.71–0.75)	0.73 (0.68–0.77)	0.76 (0.73–0.80)
T2DM	0.88 (0.86–0.90)	0.93 (0.90–0.96)	0.91 (0.89–0.93)
Mild, moderate/severe liver disease	1.38 (1.32–1.44)	1.22 (1.13–1.30)	1.31 (1.25–1.37)
Hemiplegia or paraplegia	2.03 (1.84–2.25)	2.10 (1.84–2.39)	1.95 (1.78–2.13)
Renal disease	1.10 (1.07–1.13)	1.16 (1.12–1.20)	1.12 (1.10–1.15)
Cancer and metastatic cancer	3.12 (3.04–3.21)	3.43 (3.29–3.57)	3.28 (3.20–3.37)
AIDS	1.22 (1.07–1.39)	1.14 (1.05–1.24)	1.23 (1.08–1.40)
Dialysis	2.26 (2.09–2.45)	2.98 (2.64–3.37)	2.58 (2.39–2.78)
Oxygen prior to hospitalization	1.13 (1.08–1.18)	1.15 (1.09–1.22)	1.13 (1.08–1.18)
Non-invasive mechanical ventilation	2.73 (2.59–2.88)	2.67 (2.50–2.85)	2.78 (2.65–2.91)
Invasive mechanical ventilation	7.34 (6.98–7.73)	7.70 (7.13–8.32)	7.46 (7.10–7.83)
2017	0.98 (0.95–1.01)	0.97 (0.93–1.00)	0.98 (0.95–1.00)
2018	0.94 (0.91–0.97)	0.92 (0.89–0.96)	0.93 (0.90–0.95)
2019	0.88 (0.85–0.91)	0.85 (0.82–0.89)	0.86 (0.84–0.89)
Men	NA	NA	1.13 (1.10–1.15)

COPD: chronic obstructive pulmonary disease; T2DM: type 2 diabetes mellitus; AIDS: Acquired immune deficiency syndrome. NA: not applicable.

**Table 6:** Multivariable analysis of factors associated with in-hospital mortality in people who developed hospital-acquired pneumonia (HAP), according to sex.

	MEN	WOMEN	BOTH
	OR (95% CI)	OR (95% CI)	OR (95% CI)
20–59 years old	1	1	1
60–74 years old	1.73 (1.60–1.88)	1.56 (1.37–1.76)	1.64 (1.53–1.76)
75–84 years old	2.27 (2.08–2.47)	2.03 (1.79–2.30)	2.13 (1.98–2.29)
≥85 years old	2.50 (2.25–2.77)	2.27 (1.98–2.59)	2.35 (2.17–2.55)
Myocardial infarction	1.13 (1.03–1.25)	NS	1.12 (1.03–1.22)
Congestive heart failure	1.25 (1.16–1.33)	1.37 (1.25–1.49)	1.28 (1.21–1.35)
Cerebrovascular disease	1.36 (1.26–1.47)	1.46 (1.31–1.62)	1.39 (1.31–1.48)
Dementia	1.19 (1.04–1.37)	NS	1.11 (1.00–1.23)
Mild, moderate/severe liver disease	1.47 (1.34–1.60)	1.54 (1.32–1.79)	1.50 (1.39–1.62)
Cancer and metastatic cancer	1.65 (1.55–1.76)	1.92 (1.73–2.12)	1.74 (1.64–1.84)
Undergone surgery	0.74 (0.70–0.79)	0.73 (0.67–0.80)	0.74 (0.70–0.78)
Dialysis	3.00 (2.72–3.32)	3.23 (2.74–3.81)	3.05 (2.80–3.33)
Oxygen prior to hospitalization	NS	NS	1.15 (1.01–1.32)
2017	1.04 (0.96–1.13)	0.91 (0.82–1.02)	0.99 (0.93–1.06)
2018	1.01 (0.93–1.09)	0.90 (0.81–1.01)	0.97 (0.91–1.03)
2019	0.93 (0.86–1.01)	0.85 (0.76–0.95)	0.90 (0.84–0.96)
VAP	1.90 (1.75–2.07)	2.07 (1.82–2.35)	1.95 (1.82–2.10)
Men	NA	NA	1.05 (1.01–1.10)

VAP: ventilator-associated pneumonia. NS: not significant. NA: not applicable.

## Data Availability

According to the contract signed with the Spanish Ministry of Health and Social Services, which provided access to the databases from the Spanish Register of Specialized Care-Basic Minimum Database (RAE-CMBD), we cannot share the databases with any other investigator, and we have to destroy the databases once the investigation has concluded. Consequently, we cannot upload the databases to any public repository. However, any investigator can apply for access to the databases by filling out the questionnaire available at http://www.mscbs.gob.es/estadEstudios/estadisticas/estadisticas/estMinisterio/SolicitudCMBDdocs/Formulario_Peticion_Datos_CMBD.pdf (accessed on 18 September 2021). All other relevant data are included in the paper.

## Supplementary Materials

The following are available online at https://www.mdpi.com/article/10.3390/jcm10194339/s1, Table S1: International Classification of Diseases-10 (ICD-10) codes for diagnosis and therapeutic procedures used in this investigation, Table S2: Distribution of pneumonia pathogens in patients hospitalized with community-acquired pneumonia (CAP) in Spain from 2016 to 2019, according to sex, Table S3: Distribution of pneumonia pathogens in patients who developed hospital-acquired pneumonia (HAP) in Spain from 2016 to 2019, according to sex, Table S4: Distribution of pneumonia pathogens in women and men hospitalized with community-acquired pneumonia (CAP) in Spain (2016–2019), before and after propensity score matching (PSM), Table S5: Distribution of pneumonia pathogens in women and men who developed hospital acquired pneumonia (HAP) in Spain (2016–2019), before and after propensity score matching (PSM).

## References

[1] Welte T., Torres A., Nathwani D. (2012). Clinical and economic burden of community-acquired pneumonia among adults in Europe. Thorax.

[2] Theilacker C., Sprenger R., Leverkus F., Walker J., Häckl D., von Eiff C., Schiffner-Rohe J. (2021). Population-based incidence and mortality of community-acquired pneumonia in Germany. PLoS ONE.

[3] Caroline L., Trotter C.L., Stuart J.M., George R., Miller E. (2008). Increasing hospital admissions for pneumonia, England. Emerg Infect Dis..

[4] Torres A., Barberán J., Ceccato A., Martin-Loeches I., Ferrer M., Menéndez R., Rigau D. (2020). Hospital-acquired pneumonia. Spanish Society of Pulmonology and Thoracic Surgery (SEPAR) guidelines. 2019 update. Arch Bronconeumol..

[5] de Miguel-Díez J., López-de-Andrés A., Hernández-Barrera V., Jiménez-Trujillo I., Méndez-Bailón M., Miguel-Yanes J.M., Del Rio-Lopez B., Jiménez-García R. (2017). Decreasing incidence and mortality among hospitalized patients suffering a ventilator-associated pneumonia: Analysis of the Spanish national hospital discharge database from 2010 to 2014. Medicine.

[6] Munro S.C., Baker D., Giuliano K.K., Sullivan S.C., Haber J., Jones B.E., Crist M.B., Nelson R.E., Carey E., Lounsbury O. (2021). Nonventilator hospital-acquired pneumonia: A call to action. Infect Control Hosp. Epidemiol..

[7] Torres A., Niederman M.S., Chastre J., Ewig S., Fernandez-Vandellos P., Hanberger H., Kollef M., Li Bassi G., Luna C.M., Martin-Loeches I. (2017). International ERS/ESICM/ESCMID/ALAT guidelines for the management of hospital-acquired pneumonia and ventilator-associated pneumonia: Guidelines for the management of hospital-acquired pneumonia (HAP)/ventilator-associated pneumonia (VAP) of the European Respiratory Society (ERS), European Society of Intensive Care Medicine (ESICM), European Society of Clinical Microbiology and Infectious Diseases (ESCMID) and Asociación Latinoamericana del Tórax (ALAT). Eur. Respir. J..

[8] Campogiani L., Tejada S., Ferreira-Coimbra J., Restrepo M.I., Rello J. (2020). Evidence supporting recommendations from international guidelines on treatment, diagnosis, and prevention of HAP and VAP in adults. Eur. J. Clin. Microbiol. Infect Dis..

[9] Alsawas M., Wang Z., Murad M.H., Yousufuddin M. (2019). Gender disparities among hospitalised patients with acute myocardial infarction, acute decompensated heart failure or pneumonia: Retrospective cohort study. BMJ Open.

[10] Crabtree T.D., Pelletier S.J., Gleason T.G., Pruett T.L., Sawyer R.G. (1999). Gender-dependent differences in outcome after the treatment of infection in hospitalized patients. JAMA.

[11] Colbert J.F., Traystman R.J., Poisson S.N., Herson P.S., Ginde A.A. (2016). Sex-related differences in the risk of hospital-acquired sepsis and pneumonia post-acute ischemic stroke. J. Stroke Cerebrovasc Dis..

[12] Barbagelata E., Cillóniz C., Dominedò C., Torres A., Nicolini A., Solidoro P. (2020). Gender differences in community-acquired pneumonia. Minerva Med..

[13] López-de-Andrés A., Albaladejo-Vicente R., de Miguel-Diez J., Hernández-Barrera V., Ji Z., Zamorano-León J.J., Lopez-Herranz M., Carabantes Alarcon D., Jimenez-Garcia R. (2021). Gender differences in incidence and in-hospital outcomes of community-acquired, ventilator-associated and nonventilator hospital-acquired pneumonia in Spain. Int. J. Clin. Pract..

[14] Ministerio de Sanidad Consumo y Bienestar Social. Spanish Register of Specialized Care-Basic Minimum Database [Registro de Actividad de Atención Especializada. RAE-CMBD].

[15] de Miguel-Yanes J.M., Jiménez-García R., Hernandez-Barrera V., de Miguel-Díez J., Muñoz-Rivas N., Méndez-Bailón M., Pérez-Farinós N., López-Herranz M., Lopez-de-Andres A. (2021). Sex differences in the incidence and outcomes of acute myocardial infarction in Spain, 2016–2018: A matched-pair analysis. J. Clin. Med..

[16] Instituto Nacional de Estadística Population Estimates. https://www.ine.es./dyngs/INEbase/es/operacion.htm?c=Estadistica_C&cid=1254736176951&menu=ultiDatos&idp=1254735572981.

[17] Sundararajan V., Henderson T., Perry C., Muggivan A., Quan H., Ghali W.A. (2004). New ICD-10 version of the Charlson comorbidity index predicted in-hospital mortality. J. Clin. Epidemiol..

[18] Quan H., Sundararajan V., Halfon P., Fong A., Burnand B., Luthi J.C., Saunders L.D., Beck C.A., Feasby T.E., Ghali W.A. (2005). Coding algorithms for defining comorbidities in ICD-9-CM and ICD-10 administrative data. Med. Care..

[19] Austin P.C. (2011). Comparing paired vs non-paired statistical methods of analyses when making inferences about absolute risk reductions in propensity-score matched samples. Stat. Med..

[20] Ministerio de Sanidad, Consumo y Bienestar Social Solicitud de Extracción de Datos—Extraction request (Spanish National Hospital Discharge Database). https://www.mscbs.gob.es/estadEstudios/estadisticas/estadisticas/estMinisterio/SolicitudCMBDdocs/2018_Formulario_Peticion_Datos_RAE_CMBD.pdf.

[21] Quan T.P., Fawcett N.J., Wrightson J.M., Finney J., Wyllie D., Jeffery K., Jones N., Shine B., Clarke L., Crook D. (2016). Increasing burden of community-acquired pneumonia leading to hospitalisation, 1998-2014. Thorax.

[22] Nagano H., Takada D., Shin J.H., Morishita T., Kunisawa S., Imanaka Y. (2021). Hospitalization of mild cases of community-acquired pneumonia decreased more than severe cases during the COVID-19 pandemic. Int. J. Infect Dis..

[23] Rivero-Calle I., Pardo-Seco J., Aldaz P., Vargas D.A., Mascarós E., Redondo E., Díaz-Maroto J.L., Linares-Rufo M., Fierro-Alacio M.J., Gil A. (2016). Incidence and risk factor prevalence of community-acquired pneumonia in adults in primary care in Spain (NEUMO-ES-RISK project). BMC Infect Dis..

[24] Jain S., Self W.H., Wunderink R.G., Fakhran S., Balk R., Bramley A.M., Reed C., Grijalva C.G., Anderson E.J., Courtney D.M. (2015). Community-acquired pneumonia requiring hospitalization among U.S. adults. N. Engl. J. Med..

[25] Martinez-Huedo M.A., Lopez-De-Andrés A., Mora-Zamorano E., Hernández-Barrera V., Jiménez-Trujillo I., Zamorano-Leon J.J., Jiménez-García R. (2020). Decreasing influenza vaccine coverage among adults with high-risk chronic diseases in Spain from 2014 to 2017. Hum. Vaccin Immunother..

[26] Ego A., Preiser J.C., Vincent J.L. (2015). Impact of diagnostic criteria on the incidence of ventilator-associated pneumonia. Chest.

[27] Cook A., Norwood S., Berne J. (2010). Ventilator-associated pneumonia is more common and of less consequence in trauma patients compared with other critically ill patients. J. Trauma.

[28] Dananche C., Vanhems P., Machut A., Aupee M., Bervas C., L’Heriteau F., Lepape A., Lucet J.C., Stoeckel V., Timsit J.F. (2018). Trends of incidence and risk factors of ventilator-associated pneumonia in elderly patients admitted to French ICUs between 2007 and 2014. Crit Care Med..

[29] Papazian L., Klompas M., Luyt C.E. (2020). Ventilator-associated pneumonia in adults: A narrative review. Intensive Care Med..

[30] Giuliano K.K., Baker D., Quinn B. (2018). The epidemiology of nonventilator hospital-acquired pneumonia in the United States. Am. J. Infect Control.

[31] Quinn B., Baker D.L., Cohen S., Stewart J.L., Lima C.A., Parise C. (2014). Basic nursing care to prevent nonventilator hospital-acquired pneumonia. J. Nurs. Scholarsh..

[32] Stolbrink M., McGowan L., Saman H., Nguyen T., Knightly R., Sharpe J., Reilly H., Jones S., Turner A.M. (2014). The early mobility bundle: A simple enhancement of therapy which may reduce incidence of hospital-acquired pneumonia and length of hospital stay. J. Hosp. Infect..

[33] Jiao J., Yang X.Y., Li Z., Zhao Y.W., Cao J., Li F.F., Liu Y., Liu G., Song B.Y., Jin J.F. (2019). Incidence and related factors for hospital-acquired pneumonia among older bedridden patients in China: A hospital-based multicenter registry data based study. Front. Public Health.

[34] Centers for Disease Control and Prevention, National Center for Health Statistics Pneumonia. https://www.cdc.gov/nchs/fastats/pneumonia.htm.

[35] Strassle P.D., Sickbert-Bennett E.E., Klompas M., Lund J.L., Stewart P.W., Marx A.H., DiBiase L.M., Weber D.J. (2020). Incidence and risk factors of non-device-associated pneumonia in an acute-care hospital. Infect Control Hosp. Epidemiol..

[36] de Miguel-Díez J., Jiménez-García R., Hernández-Barrera V., Jiménez-Trujillo I., de Miguel-Yanes J.M., Méndez-Bailón M., López-de-Andrés A. (2017). Trends in hospitalizations for community-acquired pneumonia in Spain: 2004 to 2013. Eur. J. Intern. Med..

[37] de-Miguel-Díez J., Jiménez-García R., Hernández-Barrera V., Zamorano-Leon J.J., Villanueva-Orbaiz R., Albaladejo-Vicente R., López-de-Andrés A. (2020). Trends in mechanical ventilation use and mortality over time in patients receiving mechanical ventilation in Spain from 2001 to 2015. Eur. J. Intern. Med..

[38] de Miguel-Díez J., López-de-Andrés A., Hernández-Barrera V., Jiménez-Trujillo I., Méndez-Bailón M., de Miguel-Yanes J.M., Jiménez-García R. (2017). Impact of COPD on outcomes in hospitalized patients with community-acquired pneumonia: Analysis of the Spanish national hospital discharge database (2004-2013). Eur. J. Intern. Med..

[39] López-de-Andrés A., de Miguel-Díez J., Jiménez-Trujillo I., Hernández-Barrera V., de Miguel-Yanes J.M., Méndez-Bailón M., Pérez-Farinós N., Salinero-Fort M.Á., Jiménez-García R. (2017). Hospitalisation with community-acquired pneumonia among patients with type 2 diabetes: An observational population-based study in Spain from 2004 to 2013. BMJ Open.

[40] Jiang H.L., Chen H.X., Liu W., Fan T., Liu G.J., Mao B. (2015). Is COPD associated with increased mortality and morbidity in hospitalized pneumonia? A systematic review and meta-analysis. Respirology.

[41] McAlister F.A., Majumdar S.R., Blitz S., Rowe B.H., Romney J., Marrie T.J. (2005). The relation between hyperglycemia and outcomes in 2,471 patients admitted to the hospital with community-acquired pneumonia. Diabetes Care..

[42] López-de-Andrés A., Perez-Farinos N., de Miguel-Díez J., Hernández-Barrera V., Jiménez-Trujillo I., Méndez-Bailón M., de Miguel-Yanes J.M., Jiménez-García R. (2019). Type 2 diabetes and postoperative pneumonia: An observational, population-based study using the Spanish Hospital Discharge Database, 2001–2015. PLoS ONE.

[43] Chughtai M., Gwam C.U., Mohamed N., Khlopas A., Newman J.M., Nadhim A., Shaffiy S., Mont M.A. (2017). The epidemiology and risk factors for postoperative pneumonia. J. Clin. Med. Res..

[44] Vallecoccia M.S., Dominedò C., Cutuli S.L., Martin-Loeches I., Torres A., de Pascale G. (2020). Is ventilated hospital-acquired pneumonia a worse entity than ventilator-associated pneumonia?. Eur. Respir. Rev..

[45] Melsen W.G., Rovers M.M., Koeman M., Bonten M.J.M. (2011). Estimating the attributable mortality of ventilator-associated pneumonia from randomized prevention studies. Crit Care Med..

