# Supplementary material for: Incidence, Outcomes and Sex-Related Disparities in Pneumonia: A Matched-Pair Analysis with Data from Spanish Hospitals (2016–2019)
Source: J Clin Med. 2021 Sep 23;10(19):4339. doi: 10.3390/jcm10194339 (PMC8509552; doi:10.3390/jcm10194339)
Supplement: Supplementary file 1 [file jcm-10-04339-s001.zip › jcm-1387978-supplementary.pdf]

Table S1. International Classification of Diseases-10 (ICD-10) codes for diagnosis and therapeutic procedures used in this investigation.

		ICD-10 codes
Community-acquired pneumonia*		J12 to J18 as primary diagnosis with a POA indicator of "Y".<br>J12 to J18 in any of the secondary diagnosis fields (2-20) and with a POA indicator of "Y".
Hospital acquired pneumonia	Not ventilator-associated pneumonia*	J12 to J18 in any diagnosis fields (2-20) and with a POA indicator coded as "N" who had a hospitalization ≥48 hours
	Ventilator-associated pneumonia*	J95.851 in any diagnosis fields (2-20) and a POA indicator coded as "N"
Bronchial fibroscopy		0BJ08ZZ
Computerized axial tomography of thorax		BW24
Dialysis		5A1D00Z, 5A1D60Z,3E1M39Z
Oxygen prior to hospitalization		Z99.81
Non-invasive mechanical ventilation		5A09357, 5A09457, 5A09557
Invasive mechanical ventilation		5A1945Z, 5A1955Z, 5A1935Z
Aspergillus		B44.9
Candida		B37.1
Escherichia coli		J15.5
Haemophilus influenzae		J14
Klebsiella pneumoniae		J15
Legionella		A48.1
Non specified *Streptococcus*		J15.4
Other gram-negative bacteria		J15.6
Influenza virus		J09.X1, J10.00 J10.01, J10.08, J11.0, J11.00, J11.08
Other virus		J12.XX
Pseudomonas aeruginosa		J15.1
Staphylococcus aureus		J15.211 AND J15.212
Streptococcus pneumoniae		J13

*Each discharge diagnosis has a "Present on Admission (POA)" indicator assigned according to the ICD-10-CM Official Guidelines for Coding and Reporting (**https://icdlist.com/icd-10/guidelines/**). The reporting options and definitions for POA are "Y" (present at admission); "N" (not present at admission); "U" (lack documentation to determine presence at admission); "W" (provider is unable to clinically determine if the condition was present); and unreported/not used.

Table S2. Distribution of pneumonia pathogens in patients hospitalized with community-acquired pneumonia (CAP) in Spain from 2016 to 2019, according to sex.

		**2016**	**2017**	**2018**	**2019**	**p-value**
Aspergillus, n (%)	Men	67(0.1)	85(0.11)	87(0.11)	70(0.09)	0.398
	Women	121(0.18)	165(0.22)	167(0.2)	146(0.18)	0.196
Candida, n (%)	Men	385(0.56)	394(0.53)	411(0.5)	402(0.5)	0.254
	Women	526(0.77)	617(0.83)	777(0.94)	776(0.97)	<0.001
Escherichia coli, n (%)	Men	381(0.56)	453(0.61)	476(0.57)	522(0.65)	0.083
	Women	44(0.06)	61(0.08)	92(0.11)	54(0.07)	0.004
Haemophilus influenzae, n (%)	Men	196(0.29)	212(0.29)	257(0.31)	259(0.32)	0.454
	Women	487(0.71)	538(0.72)	540(0.65)	579(0.72)	0.248
Klebsiella pneumoniae, n (%)	Men	932(1.36)	907(1.22)	1027(1.24)	1010(1.26)	0.071
	Women	4978(7.29)	6094(8.2)	7979(9.63)	8659(10.81)	<0.001
Legionella, n (%)	Men	642(0.94)	697(0.94)	808(0.98)	842(1.05)	0.080
	Women	2240(3.28)	1783(2.4)	3842(4.64)	3534(4.41)	<0.001
Non specified *Streptococcus*, n (%)	Men	450(0.66)	442(0.59)	753(0.91)	794(0.99)	<0.001
	Women	33(0.07)	27(0.05)	36(0.06)	28(0.05)	0.458
Other gram-negative bacteria, n (%)	Men	67(0.15)	67(0.13)	63(0.11)	65(0.11)	0.303
	Women	115(0.25)	148(0.29)	187(0.32)	172(0.3)	0.216
Pseudomonas aeruginosa, n (%)	Men	299(0.65)	367(0.71)	434(0.74)	461(0.81)	0.021
	Women	131(0.28)	171(0.33)	180(0.31)	194(0.34)	0.358
Streptococcus pneumoniae, n (%)	Men	15(0.03)	17(0.03)	27(0.05)	32(0.06)	0.179
	Women	85(0.18)	101(0.2)	144(0.24)	135(0.24)	0.089
Staphylococcus aureus, n (%)	Men	179(0.39)	183(0.35)	200(0.34)	264(0.47)	0.003
	Women	302(0.66)	381(0.74)	377(0.64)	397(0.7)	0.204
Influenza virus, n (%)	Men	3476(7.55)	4404(8.53)	6057(10.29)	6514(11.49)	<0.001
	Women	289(0.63)	308(0.6)	389(0.66)	365(0.64)	0.585
Other virus, n (%)	Men	1799(3.91)	1570(3.04)	3327(5.65)	3084(5.44)	<0.001
	Women	410(0.89)	442(0.86)	595(1.01)	788(1.39)	<0.001

Table S3. Distribution of pneumonia pathogens in patients who developed hospital-acquired pneumonia (HAP) in Spain from 2016 to 2019, according to sex.

		2016	2017	2018	2019	p-value
Aspergillus, n (%)	Men	16(0.28)	17(0.28)	14(0.21)	21(0.31)	0.681
	Women	13(0.44)	9(0.28)	7(0.2)	9(0.25)	0.286
Candida, n (%)	Men	41(0.73)	52(0.84)	51(0.75)	59(0.88)	0.757
	Women	20(0.68)	18(0.55)	21(0.59)	30(0.83)	0.496
Escherichia coli, n (%)	Men	138(2.45)	107(1.74)	142(2.09)	116(1.72)	0.011
	Women	33(1.12)	40(1.23)	36(1.01)	38(1.05)	0.823
Haemophilus influenzae, n (%)	Men	87(1.55)	79(1.28)	106(1.56)	85(1.26)	0.295
	Women	29(0.99)	29(0.89)	34(0.95)	42(1.16)	0.710
Klebsiella pneumoniae, n (%)	Men	206(3.66)	187(3.03)	204(3.01)	238(3.53)	0.086
	Women	53(1.81)	62(1.91)	62(1.73)	84(2.31)	0.289
Legionella, n (%)	Men	4(0.07)	5(0.08)	4(0.06)	10(0.15)	0.318
	Women	0(0)	0(0)	4(0.11)	0(0)	0.012
Non specified *Streptococcus*, n (%)	Men	33(0.59)	27(0.44)	26(0.38)	32(0.47)	0.412
	Women	17(0.58)	2(0.06)	12(0.34)	17(0.47)	0.004
Other gram-negative bacteria, n (%)	Men	205(3.64)	234(3.8)	247(3.64)	255(3.78)	0.944
	Women	59(2.01)	67(2.06)	55(1.54)	71(1.96)	0.357
Pseudomonas aeruginosa, n (%)	Men	292(5.19)	314(5.1)	314(4.63)	317(4.7)	0.372
	Women	115(3.92)	104(3.2)	100(2.79)	125(3.44)	0.083
Streptococcus pneumoniae, n (%)	Men	128(2.28)	161(2.61)	197(2.91)	200(2.97)	0.074
	Women	53(1.81)	63(1.94)	92(2.57)	94(2.59)	0.054
Staphylococcus aureus, n (%)	Men	164(2.92)	194(3.15)	219(3.23)	230(3.41)	0.470
	Women	68(2.32)	93(2.86)	98(2.74)	88(2.42)	0.471
Influenza virus, n (%)	Men	70(1.24)	39(0.63)	91(1.34)	80(1.19)	0.001
	Women	34(1.16)	25(0.77)	67(1.87)	53(1.46)	0.001
Other virus, n (%)	Men	29(0.52)	31(0.5)	51(0.75)	45(0.67)	0.207
	Women	18(0.61)	18(0.55)	24(0.67)	36(0.99)	0.132

Table S4. Distribution of pneumonia pathogens in women and men hospitalized with community-acquired pneumonia (CAP) in Spain (2016-19), before and after propensity score matching (PSM).

	Before PSM			After PSM		
	**MEN**	**WOMEN**	**p-value**	**MEN**	**WOMEN**	**p-value**
Aspergillus, n (%)	309(0.1)	124(0.06)	<0.001	197(0.09)	124(0.06)	<0.001
Candida, n (%)	599(0.2)	262(0.12)	<0.001	423(0.2)	262(0.12)	<0.001
Escherichia coli, n (%)	1592(0.52)	622(0.29)	<0.001	991(0.46)	622(0.29)	<0.001
Haemophilus influenzae, n (%)	2696(0.88)	1561(0.73)	<0.001	1672(0.78)	1561(0.73)	<0.001
Klebsiella pneumoniae, n (%)	1832(0.6)	676(0.32)	<0.001	1210(0.57)	676(0.32)	<0.001
Legionella, n (%)	251(0.08)	91(0.04)	<0.001	203(0.1)	91(0.04)	<0.001
Non specified *Streptococcus*, n (%)	924(0.3)	465(0.22)	<0.001	675(0.32)	465(0.22)	<0.001
Other gram-negative bacteria, n (%)	2144(0.7)	826(0.39)	<0.001	1358(0.64)	826(0.39)	<0.001
Pseudomonas aeruginosa, n (%)	3876(1.27)	1457(0.68)	<0.001	2218(1.04)	1457(0.68)	<0.001
Streptococcus pneumoniae, n (%)	27710(9.07)	20451(9.59)	<0.001	19928(9.34)	20451(9.59)	<0.001
Staphylococcus aureus, n (%)	2989(0.98)	1351(0.63)	<0.001	1974(0.93)	1351(0.63)	<0.001
Influenza virus, n (%)	11399(3.73)	9780(4.59)	<0.001	8816(4.13)	9780(4.59)	<0.001
Other virus, n (%)	2439(0.8)	2235(1.05)	<0.001	1820(0.85)	2235(1.05)	<0.001

Table S5. Distribution of pneumonia pathogens in women and men who developed hospital acquired pneumonia (HAP) in Spain (2016-19), before and after propensity score matching (PSM).

	Before PSM			After PSM		
	**MEN**	**WOMEN**	**p-value**	**MEN**	**WOMEN**	**p-value**
Aspergillus, n (%)	68(0.27)	38(0.28)	0.789	27(0.2)	38(0.28)	0.172
Candida, n (%)	203(0.8)	89(0.66)	0.136	104(0.78)	89(0.66)	0.279
Escherichia coli, n (%)	503(1.99)	147(1.1)	<0.001	278(2.07)	147(1.1)	<0.001
Haemophilus influenzae, n (%)	357(1.41)	134(1)	0.001	175(1.31)	134(1)	0.019
Klebsiella pneumoniae, n (%)	835(3.3)	261(1.95)	<0.001	432(3.22)	261(1.95)	<0.001
Legionella, n (%)	23(0.09)	4(0.03)	0.031	16(0.12)	4(0.03)	0.007
Non specified *Streptococcus*, n (%)	118(0.47)	48(0.36)	0.122	61(0.46)	48(0.36)	0.212
Other gram-negative bacteria, n (%)	941(3.72)	252(1.88)	<0.001	515(3.84)	252(1.88)	<0.001
Pseudomonas aeruginosa, n (%)	1237(4.89)	444(3.31)	<0.001	619(4.62)	444(3.31)	<0.001
Streptococcus pneumoniae, n (%)	686(2.71)	302(2.25)	0.007	336(2.51)	302(2.25)	0.173
Staphylococcus aureus, n (%)	807(3.19)	347(2.59)	0.001	433(3.23)	347(2.59)	0.002
Influenza virus, n (%)	280(1.11)	179(1.34)	0.047	157(1.17)	179(1.34)	0.227
Other virus, n (%)	156(0.62)	96(0.72)	0.244	79(0.59)	96(0.72)	0.197
